# Impact of Chemotherapy for Childhood Leukemia on Brain Morphology and Function

**DOI:** 10.1371/journal.pone.0078599

**Published:** 2013-11-12

**Authors:** Marina Genschaft, Thomas Huebner, Franziska Plessow, Vasiliki N. Ikonomidou, Nasreddin Abolmaali, Franziska Krone, Andre Hoffmann, Elisabeth Holfeld, Peter Vorwerk, Christof Kramm, Bernd Gruhn, Elisabeth Koustenis, Pablo Hernaiz-Driever, Rakesh Mandal, Meinolf Suttorp, Thomas Hummel, Chrysanthy Ikonomidou, Clemens Kirschbaum, Michael N. Smolka

**Affiliations:** 1 Klinik und Poliklinik für Kinder- und Jugendmedizin, Universitätsklinikum Carl Gustav Carus, Technische Universität Dresden, Dresden, Germany; 2 Department of Psychiatry and Neuroimaging Center, Technische Universität Dresden, Dresden, Germany; 3 Department of Psychology, Technische Universität Dresden, Dresden, Germany; 4 Department of Bioengineering, Volgenau School of Engineering, George Mason University, Fairfax, Virginia, United States of America; 5 Klinik und Poliklinik für Diagnostische Radiologie, Universitätsklinikum Carl Gustav Carus, Technische Universität Dresden, Dresden, Germany; 6 Interdisciplinary Center for Smell and Taste, Dept. of ORL, Technische Universität Dresden, Dresden, Germany; 7 Klinik für Kinder- und Jugendmedizin, Klinikum Chemnitz GmbH, Chemnitz, Germany; 8 Klinik für Kinder- und Jugendmedizin, Carl-Thieme-Klinikum Cottbus, Cottbus, Germany; 9 Department of Pediatrics, University of Magdeburg, Magdeburg, Germany; 10 University Children’s Hospital, University Medical Centre Halle, Halle, Germany; 11 Department of Pediatrics, University of Jena, Jena, Germany; 12 Pediatric Neurooncology Program, Department of Pediatric Oncology and Hematology, Charité-Universitätsmedizin Berlin, Berlin, Germany; 13 Department of Pathology, University of Wisconsin, Madison, Wisconsin, United States of America; 14 Department of Neurology, University of Wisconsin, Madison, Wisconsin, United States of America; University of Alberta, Canada

## Abstract

**Objective:**

Using multidisciplinary treatment modalities the majority of children with cancer can be cured but we are increasingly faced with therapy-related toxicities. We studied brain morphology and neurocognitive functions in adolescent and young adult survivors of childhood acute, low and standard risk lymphoblastic leukemia (ALL), which was successfully treated with chemotherapy. We expected that intravenous and intrathecal chemotherapy administered in childhood will affect grey matter structures, including hippocampus and olfactory bulbs, areas where postnatal neurogenesis is ongoing.

**Methods:**

We examined 27 ALL-survivors and 27 age-matched healthy controls, ages 15–22 years. ALL-survivors developed disease prior to their 11th birthday without central nervous system involvement, were treated with intrathecal and systemic chemotherapy and received no radiation. Volumes of grey, white matter and olfactory bulbs were measured on T1 and T2 magnetic resonance images manually, using FIRST (FMRIB’s integrated Registration and Segmentation Tool) and voxel-based morphometry (VBM). Memory, executive functions, attention, intelligence and olfaction were assessed.

**Results:**

Mean volumes of left hippocampus, amygdala, thalamus and nucleus accumbens were smaller in the ALL group. VBM analysis revealed significantly smaller volumes of the left calcarine gyrus, both lingual gyri and the left precuneus. DTI data analysis provided no evidence for white matter pathology. Lower scores in hippocampus-dependent memory were measured in ALL-subjects, while lower figural memory correlated with smaller hippocampal volumes.

**Interpretation:**

Findings demonstrate that childhood ALL, treated with chemotherapy, is associated with smaller grey matter volumes of neocortical and subcortical grey matter and lower hippocampal memory performance in adolescence and adulthood.

## Introduction

While ‘childhood cancers’ demonstrate markedly improving disease-free survival rates, we are increasingly becoming aware of the undesired ways successful treatments affect the developing brain [1]. Treatment for childhood-cancers can affect neurocognitive functions, ability to control emotions, intelligence and academic achievement [2]–[9]. Cranial irradiation and methotrexate (MTX) may cause persistent or transient white matter pathology of variable severity [10]–[14].

To achieve a 5-year event-free survival rate close to 90% in patients with acute lymphoblastic leukemia (ALL), treatment must include a mode targeting the CNS [15]. Historically, the most common methods of CNS prophylaxis are high-dose IV-MTX and intrathecal MTX, with or without craniospinal irradiation. More recently, irradiation was eliminated to prevent adverse neurologic effects and replaced by CNS-directed chemotherapy [16].

Whether the sole use of chemotherapy averts neurocognitive deficits is being debated and the results of clinical studies are conflicting [2], [8], [17], . Previous work had demonstrated that a variety of cytostatic drugs cause neuronal cell death *in vitro* at concentrations lower than those needed to exert gliotoxic effects [21]. Thus, we hypothesized that cancer chemotherapy, especially CNS-directed chemotherapy, might cause neuronal injury and affect volumes of grey matter structures. To explore this hypothesis, we designed a cross sectional clinical study with the goal to evaluate grey and white matter structure and neurocognitive functions in childhood ALL-survivors who did not receive cranial irradiation and did not develop leukoencephalopathy. In our analysis design we included brain areas with active postnatal neurogenesis (hippocampus, subventricular zone/rostral forebrain bundle/olfactory bulbs) [22], examined volumes and function of these regions in ALL-survivors and compared them to control subjects.

## Methods

### Ethics Statement

The study protocol was approved by the Ethics committee of the University of Technology, Dresden, Germany. Written informed consent was obtained from the next of kin, caretakers, or guardians on the behalf of the minors/children participants involved in the study.

### Subjects

Between March 2009 and January 2011, 27 subjects who had been treated for ALL according to either the CoALL or the ALL-BFM protocols in one of the participating centers (Dresden, Berlin, Jena, Magdeburg, Cottbus, Leipzig) were recruited. Invitations were sent to all patients who had been treated in the above centers for ALL who were 15–21 year of age at the time of the analysis. Criteria for selection were (1) ALL diagnosis prior to age 11 years, (2) no genetic syndrome, (3) no cranial irradiation, (4) no known neurologic complications of ALL treatment, (5) no known neurologic disease, (6) no ALL relapse, (7) no leukoencephalopathy on previous MRIs. Of those who responded and were willing to participate in the study, those subjects who had dental braces or other metallic parts in their bodies were excluded. None of the subjects willing to participate needed to be excluded due to acute neurologic complications during ALL treatment or leukoencephalopathy on previous MRIs. A total of 27 subjects eventually qualified to participate in the study. The control group consisted of individuals who were siblings or close friends of the ALL-subjects, were coming from similar socioeconomic environments and were invited by them to participate in the study.

Demographic details are shown in Table 1. Subjects had been stratified as low, standard or medium risk. Patients in ALL-BFM (*n = *22) received 11 injections of ITMTX in an age-dependent dose and 20,000 mg/m^2^ systemic MTX. Patients treated according to CoALL (*n = *5) had received 18 injections of ITMTX in an age-dependent dose and 3,000 mg/m^2^ systemic MTX. Systemic chemotherapy included prednisolone, vincristine, cyclophosphamide, daunorubicin, doxorubicin, asparaginase, teniposide (VM26), cytarabine, 6-mercaptopurine, 6-thioguanine, cyclophosphamide, and dexamethasone. The cumulative dose of prednisolone during induction was 1,680 mg/m^2^ in both protocols. During reinduction, patients in ALL-BFM protocol received a cumulative dose of 210 mg/m^2^ of dexamethasone. In the CoALL protocol, patients received a cumulative dose of 70–140 mg/m^2^ dexamethasone during reinduction.

**Table 1:** Demographics of ALL-survivors and non-exposed, healthy controls (N/A = non applicable, SD = standard deviation).

Variables	ALL-subjects (n = 27)	Non-exposed, healthycontrols (n = 27)	*P* Value
**Sex**			
Female	14	14	p = 1.0 (x^2^-Test)
Male	13	13	
**Race/ethnicity, n**			
White/Caucasian	27	27	
**Diagnosis**			
Pre-B-ALL	10	N/A	
c-ALL	17		
**Treatment protocol**			
CoALL	5	N/A	
ALL-BFM	22		
**Patient education, n**			
No degree	12	7	p = 0.104 (Mann-Whitney-U-Test)
High school graduate	11	12	
German Baccalaureate (Abitur)	4	8	
**Age at assessment (years)**			
Mean (SD)	17.9 (2.4)	18.3 (2.4)	p = 0.54 (t-Test)
Range	14.9–22.8	15.4–22.5	
**Age at ALL diagnosis (years)**			
Mean (SD)	5.6 (2.5)	N/A	
Range	1.1–10.2		
**Years since remission**			
Mean (SD)	12.4 (3.0)	N/A	
Range	6.1–18.5		

Subjects had been in remission for 12.5 (range 6.1–18.5) years at the time of testing. They had been treated for ALL at one of the participating institutions (University Hospitals of Berlin, Dresden, Halle, Jena, Magdeburg, and the pediatric Hospitals in Chemnitz and Cottbus, Germany). Groups were matched for age and gender.

### Structural Brain Imaging

All imaging was performed at the Neuroimaging Center at the Technische Universität Dresden on a 3.0 Tesla whole body MR-scanner (Magnetom TIM TRIO, Siemens, Erlangen, Germany) using the standard 12 channel head coil. T1-weighted three-dimensional (3-D) MPRAGE images [“Magnetization Prepared Rapid Acquisition Gradient Echo”; repetition time (TR) = 1900 ms, echo time (TE) = 2.26 ms, flip angle 9°, 256×176 mm^2^ matrix (FoV), 176 sagittal slices of 1.0 mm thickness, voxel size = 1.0×1.0×1.0 mm^3^, acquisition time: 6∶01 min:sec] were obtained.

To visualize the olfactory bulbs (OBs) a 3-D T2-weighted TSE-Sequence [“turbo spin echo”; TR = 600 ms, TE = 94 ms, flip angle 170°, 160×96 mm^2^ matrix (FoV), 96 slices of 0.5 mm thickness, acquisition time: 09∶37 min:sec] was applied with slice selection gradients oriented coronal and perpendicularly to the frontal skull base or the cribriform plate. Isotropic voxels were reconstructed at a size of 0.5 mm^3^ without interslice gap.

For further analyses all images were converted to NIfTI format and re-oriented in standard orientation.

To test whether grey matter volumes are smaller in ALL survivors we performed automated model-based segmentation with FIRST and Voxel Based Morphometry (VBM) with SPM8, respectively. Since no automated tools are available for volumetric analysis of the olfactory bulbs, this was done manually. Additionally, we conducted exploratory analyses regarding volumes of other subcortical structures with FIRST and voxel-wise whole brain analysis with VBM.

### FIRST Data Processing

We conducted a FMRIB’s Integrated Registration and Segmentation Tool-analysis [23]–[25] to explore alterations in subcortical brain regions. For this T1-data were used. Images were re-oriented in standard orientation. The Brain Extraction Tool (BET) [26], as implemented in the FSL suite was used to isolate the intracranial space from the head and neck [27]. The FIRST tool of FSL was used to segment subcortical structures, specifically left and right hippocampus, amygdala, nucleus accumbens, caudate nucleus, globus pallidus, putamen, thalamus and brain stem; the FAST tool was used to calculate total brain volume. Segmentation quality was checked visually by an experienced observer. The volume of each of these structures was measured based on the masks produced, and subsequently normalized based on the total intracranial volume resulting from BET.

### VBM Data Processing

We additionally conducted an optimized Voxel Based Morphometry-analysis (VBM-analysis) [28] to cross-validate the FIRST data and to investigate the differences in the volumes of gray matter (GM), white matter (WM), and cerebrospinal fluid (CSF) between the ALL survivors and the control group. T1-weighted MRI images were analyzed using the VBM8-toolbox (http://dbm.neuro.uni-jena.de/vbm) for SPM8 (http://www.fil.ion.ucl.ac.uk/spm) based on Matlab 7.10 (MathWorks, Ismaning, Germany). We used the standard tissue probability maps templates (ICBM) to segment GM, WM, and CSF as implemented in SPM8. The VBM preprocessing included the steps: (1) check for scanner artifacts and obvious anatomical abnormalities for each subject by a senior neuroradiologist; (2) segmentation and normalization to the SPM standard template of all individual T1-images using the Hidden Markov Random Field (HMRF) option to minimize the noise level of the segmentation. As a result of this step the GM, WM and CSF segments, as well as individual absolute GM, WM und CSF volumes were computed. (3) The modulation step is used to maintain the total amount of contraction of GM, WM, and CSF by scaling as it is in the source images; (4) Check for homogeneity across the sample followed by the standard procedure steps described in Good et al. 2001 [29]. All subject images passed this homogeneity quality check successfully, thus all images could be included in further statistical analyses. (5) For smoothing images, we used a smoothing-kernel of 8 mm for modulated tissue segments.

Finally, the smoothed, modulated and normalized data were used for the statistical analysis.

### Diffusion Tensor Imaging Analysis

Diffusion tensor imaging data (voxel size 2.4×2.4×2.4 mm^3^, 60 slices, in-plane matrix size 128×128, echo time 104 ms, b-value 1300, 32 gradient directions) were acquired on all subjects. Data were processed in FSL in order to calculate the fractional anisotropy (FA) and mean diffusivity (MD) maps. Following that, voxelwise statistical analysis of the FA data was carried out using TBSS (Tract-Based Spatial Statistics), part of FSL [26], [30]. For this analysis, subjects were divided in two groups, for chemotherapy and control. First, FA images were created by fitting a tensor model to the raw diffusion data using FDT, and then brain-extracted using BET [26]. All subjects’ FA data were then aligned into a common space using the nonlinear registration tool FNIRT [31], [32], which uses a b-spline representation of the registration warp field [33]. Next, the mean FA image was created and thinned to create a mean FA skeleton, which represents the centers of all tracts common to the group. Each subject’s aligned FA data was then projected onto this skeleton and the resulting data fed into voxelwise cross-subject statistics. Similar analysis was carried out using the MD data.

### Volumetric Analysis of the Olfactory Bulbs (OB)

Volume measurements were performed by MG and NDA in consensus by manual segmentation of the coronal slices through the OBs on both sides separately excluding the olfactory tract as previously described [34].

### Statistical Comparisons of VBM, FIRST and OBs Volumetric Data

Statistical analyses regarding group differences of absolute volumes of GM, WM, and CSF segments computed with VBM, the volumes of subcortical structures estimated with FIRST, as well as of the olfactory bulbs volumes were done using SPSS (Version 19; SPSS GmbH Software, Munich, Germany).

For analyzing the absolute volumes of GM, WM, CSF, right and left hippocampus, amygdala, nucleus accumbens, caudate nucleus, globus pallidus, putamen, thalamus and brainstem as well as total volumes of olfactory bulbs a multivariate analysis of covariance, with group as between-subject factor was conducted. The volumes of right and left olfactory bulb were subjected to statistical analysis of variance for repeated measures, with side as within-subject factor and group as between-subject factor. To evaluate effect of age and sex on brain volume, age and sex were taken as covariates for each type of analysis. The level of statistical significance was set at p≤0.05 for all analyses. Two-tailed tests were used.

To cross-validate the FIRST data a whole brain voxel-wise analysis with SPM (VBM) was performed. For this purpose the smoothed, normalized, modulated GM, WM and CSF image segments of each group were entered into separate independent-samples t-tests analysis in SPM8 with age and sex as covariates. An absolute threshold mask of 0.2 was used to prevent for partial volume effects for different tissue types. This step prevents getting results in WM although only made an analysis for GM. This effect is due to the negative correlation of GM and WM intensity values around the border between GM and WM. By choosing an absolute threshold masking statistics was only computed in those areas that are above this threshold. Areas below this threshold will be excluded. The statistical significance level for the explorative whole brain VBM analysis was set at p≤0.001 (uncorrected for multiple comparisons).

### Olfactory Testing

In all subjects a short history was taken which ascertained that they did not have serious illnesses, or acute or chronic diseases which might affect the response to chemosensory stimuli. They were instructed not to eat or to drink anything but water 1 hour prior to the measurements, to avoid chemosensory desensitization. Olfactory function was assessed monorhinally in all individuals by means of the validated “Sniffin’ Sticks” test kit which is comprised of 3 individual tests of olfactory function (phenyl ethyl alcohol odor threshold, odor discrimination, and odor identification). The scores of the individual tests were summated to the so-called “TDI-score” which is a reliable means to estimate the degree of overall olfactory function [35]. To study differences between groups statistical analysis of variance (ANOVA) for repeated measures, with side as within-subject-factor, group as between-subject-factor, age and sex as covariates was conducted (SPSS, Version 19). The statistical significance level was set at p≤0.05. For our hypothesis of reduced olfactory function two-tailed test was used.

### Neuropsychological Testing

Neuropsychological testing took place in a single session either on the day of MRI examination (separated by a resting period) or on a separate day within the following seven days. Total testing time for all neuropsychological tests was approximately 90 min.

#### Memory

Hippocampus-dependent memory functions were measured with the German learning and memory test “Lern- und Gedächtnistest” (LGT-3) [36]. This paper-and-pencil test comprises a learning period and a subsequent testing period and was applied in groups of 2–4 participants. With a total of six subtests, the LGT-3 assesses encoding and maintenance of verbal and figural material. Outcome measures are the total memory-performance score as indicator of general learning and memory ability as well as memory-performance scores for verbal and figural material, respectively, to allow for material-dependent analysis of memory functioning [36].

#### Executive functions

In order to measure mental set shifting and interference control as two pivotal executive functions, participants performed a task-switching paradigm [37] on an IBM-compatible personal computer. Stimulus presentation and data recording were realized using Presentation software (version 0.71; Neurobehavioral Systems, Inc., Albany, CA, USA). During this task, participants switched between categorizing visually presented single-digit numbers (1–9, except 5) as smaller or larger than five and odd or even by pressing either a left or right key on a QWERTZ keyboard. Task order was random, and a visual cue presented either 200 or 1000 ms prior to the target stimulus indicated the current task. Performance differences between trials in which the previous task repeated and trials in which the task switched (switch costs) were analyzed as measure of the cognitive control processes that serve to update action-relevant information. Performance differences between trials with target stimuli that require the same response in both tasks and trials with target stimuli that demand different responses, depending on the current task (target-congruency effect), reflect the amount of interference from the currently irrelevant task and were used as indicator of interference control. Each participant performed a total of 32 practice trials and 192 test trials.

#### Attention

Selective attention, sustained attention, and impulsivity were assessed with the Continuous Performance Test (CPT) [38]. In this computerized task, five letters (i.e., H, O, T, X, and Z) were presented on the screen for 200 ms each with inter-stimulus intervals of 2000 ms in pseudorandomized sequences. Participants were instructed to only respond to a particular target sequence (i.e., O followed by X) by pressing the space bar on a QWERTZ keyboard with their right index finger. 400 stimulus sequences were presented comprising 200 irrelevant sequences (i.e., neither O nor X), 100 non-target sequences (i.e., O followed by another letter than X), and 100 target sequences. The number of missing responses despite target-sequence presentation (omission errors) and the number of erroneous responses to irrelevant and non-target sequences (commission errors) were analyzed as indicators of sustained attention and selective attention as well as impulsivity, respectively. Furthermore, the latency between target-sequence presentation and the according (correct) response (response time) was calculated as measure of the time required to provide a correct response (see reference 38 for further details on the CPT).

#### Intelligence

The short version of the Culture Fair Intelligence Test (CFT) 20-R [39] was chosen to assess basic intelligence (also referred to as “general fluid ability”) with a minimum of cultural and educational bias. The paper-and-pencil test was conducted in groups of 2–4 participants resulting in a total performance score for each individual [39].

#### Data analysis

Outcome measures of the LGT-3, CPT, and CFT 20-R were transformed into standardized values (i.e., T, C, and IQ values, respectively) based on age-dependent norm data. To analyze differences between ALL-subjects and controls, *t* tests for independent measures were conducted. For the analysis of task-switching performance, the within-subjects factors task transition (switch vs. repetition), cue-target interval (200 vs. 1000 ms), and target congruency (incongruent vs. congruent) and the between-subjects factor group (ALL-subjects vs. controls) were entered into repeated-measures ANOVAs on mean response times and error rates. Due to technical problems, CPT data of two control subjects and task-switching data of one participant of the control group could not be obtained. To explore brain behavior correlations we additionally computed Pearson correlation coefficients between individual volumes of brain regions and the individual performance in the neuropsychological tests. These correlation analyses were restricted to those brain regions and performance scores that differed between patients and controls. The statistical significance level for all analyses of neuropsychological parameters was set at *p*≤0.05. Key performance outcome measures of all neuropsychological tests were conducted two-tailed.

## Results

### Structural Brain Imaging

FIRST analysis revealed significantly smaller volumes of bilateral hippocampi (Table 2), the left nucleus accumbens, amygdala and thalamus in ALL-subjects.

**Table 2:** Smaller volumes of subcortical structures in ALL-subjects compared to controls as revealed by FIRST analysis.

Brain area		ALL-subjects(n = 27) Volume in mm^3^(Mean ± SEM)	Controls (n = 27) Volumein mm^3^ (Mean ± SEM)	Group difference Volumein mm^3^ (Mean)	*P* value*
Hippocampus	L	3,635±90	3,943±90	308	0.019*
Hippocampus	R	3,794±81	3,999±81	206	0.078
Amygdala	L	1,211±46	1,368±46	158	0.019*
Amygdala	R	1,257±46	1,310±46	53	0.417
Accumbens	L	556±24	635±24	79	0.024*
Accumbens	R	460±24	460±24	0.2	0.995
Caudate	L	3,734±88	3,818±88	86	0.492
Caudate	R	3,831±86	3,918±86	87	0.477
Pallidum	L	1,659±31	1,744±31	86	0.059
Pallidum	R	1,685±29	1,764±29	79	0.062
Putamen	L	4,801±97	5,020±97	219	0.116
Putamen	R	4,875±94	5,122±94	246	0.072
Thalamus	L	7,637±138	8,136±138	499	0.014*
Thalamus	R	7,542±129	7,882±129	339	0.069
Brainstem		20,053±417	21,139±417	1,086	0.072
Total brain		1,437,177±22,182	1,477,626±22,182	40,448.49	0.204

* two-tailed.

* P<0.05 two-tailed.

**Figure 1:**
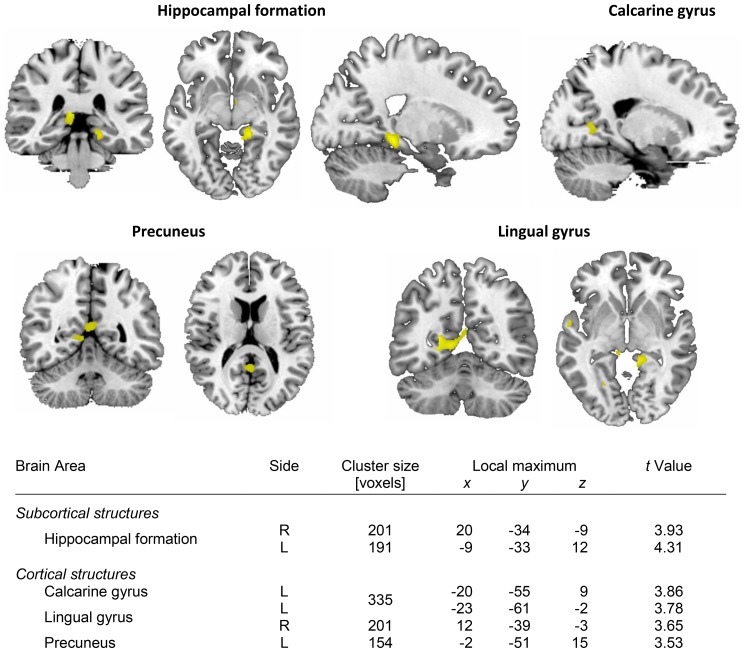
Brain areas which demonstrated decreased volume based on VBM analysis. Local maxima (MNI) correspond to slices depicted. *P* uncorrected <0.001, cluster size >20 voxels; expected false discovery rate: *P*<0.02.

Exploratory whole brain VBM analysis revealed significantly smaller volumes of bilateral hippocampi in ALL-survivors. Within cortical areas, volumes of the left calcarine gyrus, the lingual gyri and the left precuneus were significantly lower in ALL-subjects (Figure 1).

Absolute volumes of GM, WM and CSF segments (computed with SPM/VBM) and total brain volumes (computed with FIRST and SPM/VBM) did not differ significantly (Table 3). As expected, total brain, grey and white matter as well as CSF volumes in males were higher compared to females. Moreover, grey matter volumes were smaller in older participants.

**Table 3:** Results of statistical comparison of the absolute volumes of GM, WM and CSF as well as the total brain volume computed with SPM/VBM.

VBM-segments	ALL-subjects (n = 27) Volumein cm^3^ (Mean ± SEM)	Controls (n = 27) Volumein cm^3^ (Mean ± SEM)	Estimated volume differencein cm^3^ (Mean)	*P* value*
GM	687±13	700±12	16	0.22
WM	519±14	543±12	23	0.14
CSF	197±4	207±5	10	0.12
Total	1,404±29	1,450±27	49	0.12

* two-tailed.

We compared the values of total brain volume obtained by FSL and VBM - total brain volume is the only common volumetric value obtained by FSL and VBM. A highly significant correlation (0.96, *p*≤0.001) was found.

MR-images of 16 ALL-subjects and 16 controls could be used for OB volumetric analysis. Although numerically smaller OB volumes in ALL-subjects were seen, differences were not significant (Table 4).

**Table 4:** Olfactory bulb volumes.

Side	ALL-subjects (n = 16) Volumein mm (Mean ± SEM)	Controls (n = 16) Volumein mm^3^ (Mean ± SEM)	Group difference Volumein mm^3^ (Mean)	*P* value*
L	33±4	40±4		
R	30±4	38±4		
Total	63±7	78±7	15	0.18

* two-tailed = 0.179.

### DTI Analysis

Regional trends for higher fractional anisotropy (FA) and smaller medial diffusivity (MD) in ALL-subjects were observed, however they did not reach statistical significance.

### Olfactory Function

No threshold differences for smell perception and identification were observed between groups (Table 5).

**Table 5:** Results from olfactory function tests.

	ALL-subjects (n = 26)[TDI-Score] ± SEM	Controls (n = 27)[TDI-Score] ± SEM	Diff.	*P* Value*
Threshold				
R	8.3±0.6	8.3±0.6	0.02	0.98
L	7.8±0.7	8.7±0.6	1.0	0.30
Identification				
R	12.8±0.4	12.5±0.4	0.3	0.55
L	12.7±0.4	12.8±0.4	−0.1	0.79

* two-tailed.

### Neuropsychological Testing

Significant differences between groups were found for all memory-performance measures of the LGT-3, omission errors in the CPT, and total performance in the CFT 20-R, whereas no differences occurred for commission errors and response time in the CPT as well as switch costs and the target-congruency effect in task switching (Table 6).

**Table 6:** Results of neuropsychological testing.

Outcome measure	ALL-subjects (n) Mean ± SEM	Controls (n) Mean ± SEM	*P* value
***LGT-3***			
Total memory-performance score (T value)	41.0±2.5 (27)	52.2±2.2 (27)	0.002**
Verbal memory-performance score (T value)	42.2±2.0 (27)	50.0±1.8 (27)	0.005**
Figural memory-performance score (T value)	46.0±1.9 (27)	54.4±2.1 (27)	0.004**
***Task switching***			
Switch costs in response times (ms)	157.3±23.3 (27)	183.7±25.3 (26)	0.28
Switch costs in error rates (%)	3.27±1.3 (27)	1.99±0.8 (26)	0.15
Target-congruency effect in response times (ms)	100.9±25.0 (27)	86.6±14.7 (26)	0.30
Target-congruency effect in error rates (%)	9.8±1.9 (27)	7.5±1.1 (26)	0.14
***CPT***			
Omission errors (C value)	4.6±0.3 (27)	3.7±0.3 (25)	0.05*
Commission errors (C value)	4.9±0.4 (27)	4.4±0.5 (25)	0.62
Response time (C value)	5.4±0.3 (27)	5.4±0.3 (25)	0.42
***CFT 20-R***			
Total performance score (IQ value)	101.0±2.7(27)	111.0±2.8(27)	0.015*

*Note.* CFT 20-R = Culture Fair Intelligence Test 20-R. CPT = Continous Performance Test. LGT-3 = German learning and memory test “Lern- und Gedächtnistest”.

To rule out an interpretation of the revealed memory impairments in terms of attention-deficit consequences and/or generally decreased cognitive performance, we conducted analyses of covariance with both omission errors in the CPT and individual total performance score in the CFT 20-R as covariates on all three memory-performance measures. The analyses clearly confirmed the effects for total memory-performance score, *F*(1, 48) = 3.840, *p* = 0.028, and figural memory-performance score, *F*(1, 48) = 3.889, *p* = 0.027. For the verbal memory-performance score, the effect was not significant, *F*(1, 48) = 2.455, *p = *0.062.

### Correlation between Brain Area Volumes and Memory Function

The verbal memory-performance score of the LGT-3 correlated significantly (r = 0.291, p = 0.033) with the volume of the left amygdala, whereas the figural memory-performance score of the LGT-3 showed a significant positive correlation (r = 0.282, p = 0.039) with the volume of the right hippocampus. That means, the smaller the volume is the worse the memory performance.

We also detected significant negative correlations between omission errors in the CPT and the volume of the left amygdala (*r* = −0.29, *p* = 0.037) and the caudate (left, *r* = −0.356, *p* = 0.010; right, *r* = −0.313, *p* = 0.024). A negative correlation means that the lower the brain area volume is the higher is the number of errors within the attention task. No other correlations were significant.

## Discussion

In this cross sectional study we show that childhood low and standard risk ALL, successfully treated with systemic and CNS directed chemotherapy, associates with lower grey matter volumes of neocortical and subcortical structures in adolescence and young adulthood. In this study we did not detect any white matter pathology in ALL-survivors. Morphological analysis of MR images using FIRST and VBM with SPM revealed significantly smaller hippocampal volumes in ALL survivors. Exploratory analyses unraveled smaller volumes in amygdala, thalamus and nucleus accumbens. In addition, VBM demonstrated smaller grey matter volumes in the calcarine and lingual gyri and the precuneus in ALL-survivors.

In our study, lower hippocampal volumes in ALL survivors correlated with significantly lower neurocognitive performance scores compared to control subjects from similar socioeconomic backgrounds. Significant differences in hippocampus-dependent learning and memory ability, sustained attention, and basic intelligence were measured between the ALL- and control groups. Lower figural and verbal memory functioning and lower volume in hippocampus and amygdala, respectively, not only co-occurred, but were correlated, indicating a causal relation. Involvement of grey matter structures which comprise components of the limbic system, i.e. nucleus accumbens, amygdala and thalamus, raises questions about their contribution to reported psychiatric morbidities, especially decreased ability to handle stress and propensity towards depressive mood following chemotherapy [40].

Lower scores in hippocampus-dependent memory functions in ALL-survivors were also found when controlling for the observed impairment in sustained attention and lower general fluid ability. Thus, the revealed memory impairment reflects a possible domain-specific consequence of chemotherapy for ALL in childhood. Long term impact of chemotherapy on attention was highly process-specific, as sustained attention was substantially impaired while, for example, impulsivity was not found to be altered in ALL-survivors. It is interesting to note that, within the same sample, no significant differences were found for the executive functions of flexible mental set shifting and interference control. This result pattern on the cognitive functioning level fits the structural results of substantially smaller hippocampal volumes but no significant differences in prefrontal cortex volumes in ALL-survivors as compared to controls.

OB volumes were not significantly smaller in ALL-survivors, and olfactory functions were unaffected.

Our findings add to reports of other investigators on the impact of chemotherapy and irradiation on the developing human brain [10], [12]–[14], [17]. Previous studies have described white matter pathology in ALL survivors. Carey et al [17] used VBM analysis in subjects who were treated with systemic and intrathecal chemotherapy only and reported reduced white matter volumes in the right frontal lobes compared to healthy individuals. Dellani et al [10] used DTI analysis and examined the images of 13 adult survivors, 17–37 years old, who had been treated with total brain radiation and chemotherapy. These authors reported significantly reduced fractional anisotropy values in the temporal lobes, hippocampi and thalami which were accompanied by significant white matter volume loss. Reddick et al [13] used voxel based analysis of patients during treatment for ALL. Their analysis identified specific white matter tracts involving predominantly the anterior, superior and posterior corona radiata and superior longitudinal fasciculus which were at increased risk for the development of T2 weighted hyperintensities during therapy for childhood ALL. Of note is that in this study patients did not receive craniospinal irradiation.

In our analysis we did not detect differences in white matter between ALL survivors and healthy controls. One possible explanation to consider for this discrepancy is that we only studied subjects who were classified as low or standard risk and who may have suffered none or reversible white matter injury during treatment. Interestingly and in contrast to previous studies which linked cognitive deficits to white matter pathology, our study suggests that systemic and CNS directed chemotherapy for ALL may also affect grey matter and cause neurocognitive deficits that correlate with the pattern of grey matter pathology.

The question remains open as to why childhood ALL, successfully treated with chemotherapy only, associates with smaller grey matter volumes and worse hippocampal memory performance in adolescence and adulthood. Given the cross-sectional nature of this study, we can only speculate about potential pathomechanisms. The possibility of selective neuronal injury by cytostatic drugs ought to be entertained, since, as shown previously, excitotoxic and apoptotic neurodegeneration can be induced *in vitro* and *in vivo* by agents used in cancer chemotherapy [21]. Interestingly, we had previously demonstrated that cytostatic drugs are more potent neurotoxins than they are gliotoxins, which fits well with our current observation of lack of white matter pathology in ALL survivors.

Localization of smaller cortical volumes within occipital areas raises an interesting hypothesis: the posterior reversible encephalopathy syndrome (PRES) is a potential complication in immune compromised patients [41]. PRES is associated with breakdown of the blood brain barrier and resulting focal edema over parietooccipital brain regions, visible on MRI. PRES was not documented in any of the ALL-subjects who participated in this study. However, the fact that all neocortical areas with decreased grey matter volumes were localized within the predilection areas of PRES raises the speculation that mild, clinically and radiographically unrecognized forms of PRES might contribute to this cortical pathology. Opening of the blood brain barrier during chemotherapy might potentially lead to neurotoxic concentrations of cytostatic drugs within the cortex, the thalamus, amygdala and nucleus accumbens. The possibility of selective vulnerability of certain neuronal groups to the chemotherapeutic agents used for ALL treatment also ought to be entertained. Further, loss of cortical or hippocampal projections due to primary injury in those areas might have secondarily affected neuronal survival and connectivity within subcortical structures.

Smaller volumes of the amygdala, accumbens and thalamus may be contributing to emotional problems reported in ALL-survivors [40]. Similarly, decreased volumes in the lingual and calcarine gyri and the precuneus might lead to subtle deficits in visual processing and recognition memory [42].

Impairment of postnatal neurogenesis by cytostatic drugs might partly explain smaller hippocampal volumes in ALL survivors. Lack of OB volume differences between ALL-survivors and controls does not contradict the antineurogenesis hypothesis, it could in fact be explained by recent findings by Sanai et al. [43]. The subventricular zone of many adult and non-human mammals generates large numbers of new neurons destined for the olfactory bulb. Sanai et al. demonstrated that the infant human subventricular zone contains an extensive corridor of immature neurons before 18 months of age but this germinal activity subsides in older children and is nearly extinct in adulthood. Since the majority of our subjects were older than 18 months at the time of ALL diagnosis, neurogenesis in the subventricular zone targeting the OBs is expected to have been minimal to absent at the time of ALL-treatment.

There are additional limitations to our study and the conclusions drawn. Our VBM and FIRST analyses are based on T1-weighted images only. Future studies might use multispectral morphometry by combining information from T1-weighted and T2-weighted MR images which might improve segmentation of grey and white matter and thereby results of automated volumetry. There are also limitations pertaining to the impact of the smoothing kernel size on the results of lesion detection in the VBM analysis. As shown by Zhang et al [44] the optimal size of the smoothing kernel should match that of the expected morphological differences. We decided to use an 8-mm FWHM Gaussian kernel for the VBM analysis because this should be appropriate when focusing on differences in hippocampal volume. Therefore, use of additional smoothing kernels might have revealed further morphological alterations in other brain regions.

Our findings underline the importance of detailed neuropsychological follow up and timely interventions for all childhood cancer survivors, since deficits can be subtle but still sufficient to affect long term academic achievement and mental health. Of clinical concern is the finding of lower grey matter volumes within posterior brain regions, predilection areas of PRES. We suggest that screening for mild forms of PRES and early institution of appropriate treatment should be aggressively pursued even in uncomplicated cases of cancer chemotherapy in children. Prospective clinical studies are needed to confirm our observations and conclusions, define the most vulnerable age groups und explore potential underlying pathomechanisms of neurotoxicity of childhood cancers and cancer chemotherapy.
